# Characterization of effective moisture diffusivity based on pore structure of concrete

**DOI:** 10.1038/s41598-024-66300-w

**Published:** 2024-08-08

**Authors:** Osamah H. A. Dehwah, H’mida Hamidane, Yunping Xi

**Affiliations:** 1 Department of Civil, Environmental and Architectural Engineering, University of Colorado Boulder, Boulder, CO 80309 USA; 2 Laboratory of Applied Civil Engineering, Echahid Cheikh Larbi Tebessi University, 12002 Tebessa, Algeria

**Keywords:** Moisture diffusion, Diffusion mechanisms, Pore size distribution (Microstructure), Adsorption isotherm, Concrete durability, Composites, Civil engineering

## Abstract

Concrete durability is greatly influenced by the transport rate of aggressive chemicals. Moisture diffusion plays a key role in the long-term performance of cementitious materials, as it facilitates the entry of aggressive chemicals into concrete. The pore size distribution plays a critical role in determining moisture diffusivity. However, the characteristics of the concrete pore structure have not been included comprehensively in the material models so far. In this paper, a theoretical model was developed to obtain the pore size volume fractions for each diffusion mechanism including Molecular, Knudsen and Surface diffusions. An effective moisture diffusivity in concrete was then obtained using the weighted average based on the diffusion mechanisms and pore size volume fractions. The model’s validity was demonstrated by comparing model predictions with available experimental data. The findings of this study provide valuable insights into the behavior of the concrete pore structure and its impact on moisture diffusivity.

## Introduction

The durability of cementitious materials is highly dependent on the transport rate of aggressive substances^1^. It has been demonstrated widely that many deterioration processes in concrete structures can be controlled by limiting the ingress of deleterious aqueous solutions (such as water, chlorides, sulfates, etc.) or gasses (e.g., carbon dioxide)^2,3^. Moisture diffusion in concrete is an important process affecting the long-term performance of cementitious materials^4–7^. The variation of moisture inside concrete affects several of its long-term properties, such as creep and shrinkage, which subsequently affect the mechanical properties such as compressive strength and elastic modulus. In addition, the moisture distribution within concrete greatly influences the resistance to the fire or freezing–thawing induced damage^8^.

The moisture transport properties and the pore size distribution of concrete are two important and interrelated characteristics^9^. The moisture transport inside cementitious materials is basically controlled by the microstructure of the material, and especially by the pore size distribution^9,10^. The pore-structure of cement paste varies in dimension spanning from angstrom to micrometer and further up to millimeter scale. As stated by Powers and Brownyard^11^ and Neville^12^, the pores of cement paste can be roughly divided into gel pores (several nanometers), capillary pores (several nanometers to several micrometers) and isolated air voids with sizes ranging from nanometers to millimeters. The whole range of pore-structure has been investigated by using different experimental techniques^10,13–15^. One of the most popular experimental measurement methods to determine the pore structure is the mercury intrusion porosimetry (MIP)-ISO 15,901^16–20^. Another method is based on adsorption isotherms, several empirical models were developed to determine the pore size distribution using the results from adsorption isotherms^9,21^. Empirical models were also proposed for the adsorption isotherms of concrete by Giarma^22^ and Xi et al.^4^. There are several key factors influencing the formation of microstructure of cement paste such as water-to-cement ratio, type of cement, curing time, and temperature. All of these parameters are considered in the models for adsorption isotherms of cement paste^4^.

Various diffusion mechanisms coexist in the cement paste because of the diminutive size of the mean free path of water vapor and the wide range of pore sizes^23^. The diffusion mechanisms that may be involved in concrete are molecular diffusion, Knudsen diffusion and surface diffusion. The diffusion mechanisms do not operate in the same range of pore sizes. Therefore, in a specific size of pores, there is a dominant diffusion mechanism. Each individual diffusion mechanism of moisture in vapor and gaseous media has been studied over a long period of time^24–27^. Wilke and Chang^28^ developed an empirical expression for the molecular diffusion (W–C relation), and then many researchers improved the W–C relation^29,30^. Richard David^31^ developed a formula for the Knudsen diffusion mechanism. A model for the surface diffusion was developed based on the adsorption theory using the Langmuir isotherm^32^. There is a lack of theoretical models linking the pore size distribution to the moisture diffusivity of concrete specifically including the different diffusion mechanisms that occur in nanoscale pores. Therefore, this paper aims to develop a theoretical model for the effective moisture diffusivity of concrete based on all of the controlling moisture diffusion mechanisms i.e., Molecular, Knudsen and Surface and considering the entire internal pore size distribution of cement paste.

First, the adsorption isotherm of concrete will be explained, and a model for adsorption isotherms of concrete will be introduced taking into account the type of Portland cement, the water to cement ratio, the curing time, and the temperature as well as the relative humidity level inside pores. The model for adsorption isotherms will be used to determine the pore size distribution of cement paste and concrete. Second, the pore size distribution will be divided into three ranges based on the controlling diffusion mechanism in each range of the pore sizes. The proposed model will be the volumetric average of the three controlling mechanisms, and the weighted parameters are based on the pore size fraction of each mechanism. Finally, the proposed model for the effective diffusivity of concrete is implemented in a finite element code, and the numerical simulation results are validated with available test data.

## Theoretical transport model for cementitious materials

### Moisture diffusion equation

Fick’s law is used to find the moisture flux ($J$) in concrete as demonstrated in Eq. (1). The mass balance equation can be expressed as illustrated in Eq. (2); Simplified form of the mass balance equation can be found in Eq. (3) ^8,33^.

1 $$J = - D_{h} {\text{grad}} H$$

2 $$\frac{\partial W}{{\partial t}} = \frac{\partial W}{{\partial H}}\frac{\partial H}{{\partial t}} = - {\text{div}}\left( J \right)$$

3 $$\frac{\partial W}{{\partial H}}\frac{\partial H}{{\partial t}} = \nabla \cdot \left( {D_{h} \nabla H} \right)$$

where $D_{h}$ and $H$ are the humidity diffusivity coefficient and the pore relative humidity respectively; $W$ is the total water content for unit volume of material; $t$ is the time; $\frac{\partial W}{{\partial H}}$ is the moisture capacity^4,34^.

### Moisture diffusion through porous media

**Figure 1:**
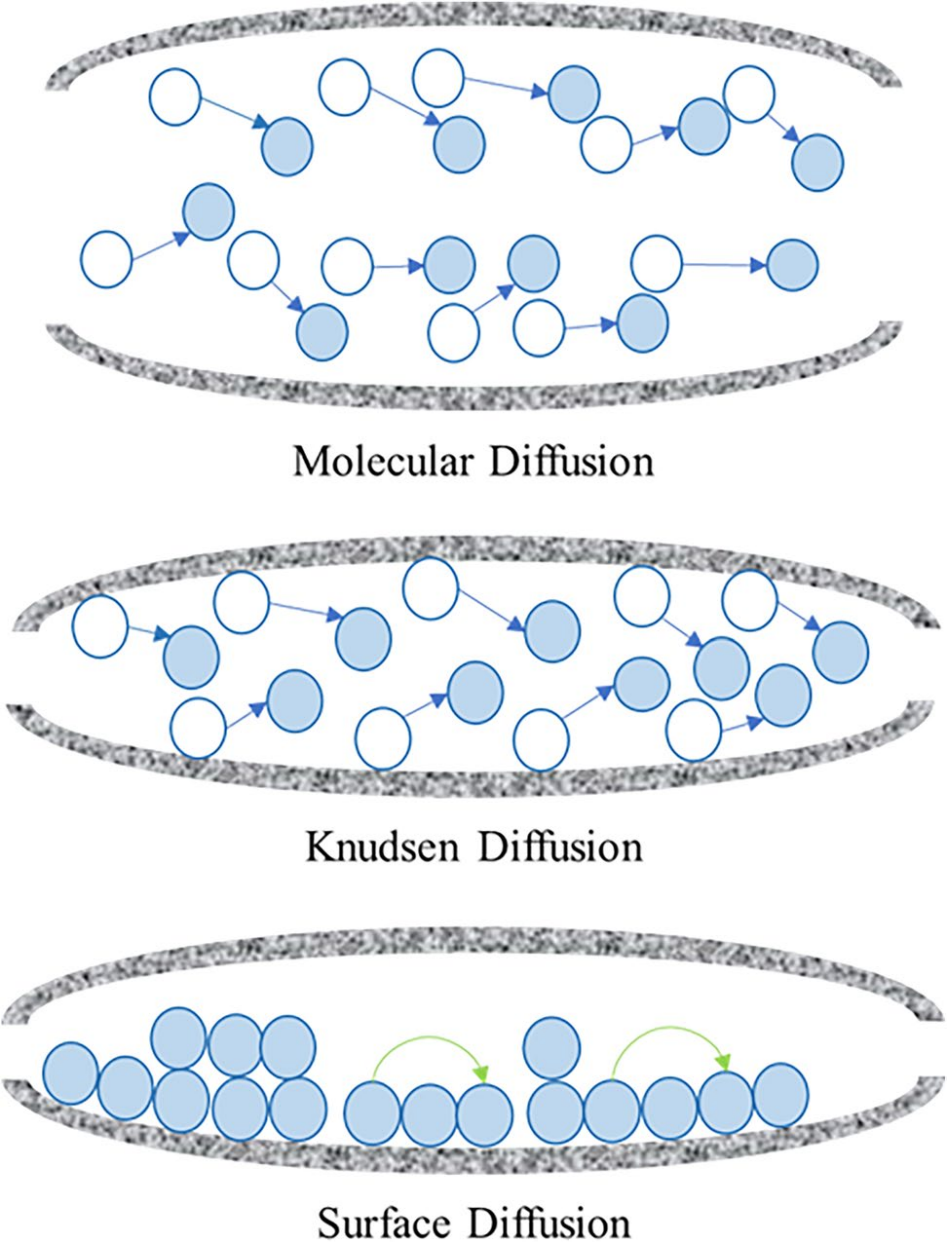
Diffusion mechanisms in cementitious materials.

The diffusion of moisture in concrete is controlled by various diffusion mechanisms. The controlling diffusion mechanism is primarily influenced by the pore structure of concrete. In reality, the transport mechanisms of moisture are considered to be extremely complex due to many factors such as degree of saturation (moisture content in the porous network), the degree of hydration process of Portland cement, and the randomness of porous structure in concrete^4,23,35,36^. For straight cylindrical pores of different sizes which have been used as a simplified pore structure, three types of transport mechanisms have been considered which are Molecular diffusion (Ordinary diffusion), Knudsen diffusion and Surface diffusion^23,36–38^, and they can operate singularly or simultaneously depending on the pore sizes^4,23,36,39^. Therefore, the prediction of the total diffusivity depends on the details of the pore structure of concrete. Figure 1 shows the three transport mechanisms.

The Molecular diffusion (ordinary diffusion) dominates when the diameter of the pore size is larger than the mean free path of water vapor. The moisture molecules move with collisions against each other. The molecular diffusion of liquids can be obtained using the modified Wilke–Chang equation^40^:

1. 4 $$D_{M} = 7.4 \times 10^{ - 8} \frac{{T\left( {XM} \right)^{1/2} }}{{\mu V_{b}^{0.6} }}$$
  where $D_{M}$ is the molecular diffusivity in ${\text{cm}}^{2} /{\text{s}}$; $T$ is the temperature in $K$; $X$ is an empirical “association constant”, X = 2.6^29^; $M$ is the molecular weight; $\mu$ is the viscosity; $V_{b}$ is the molar volume. The mean free path (MFP) of water vapor can be calculated using Eq. (5), it is approximately $86{\text{ nm}}$^41,42^.
  5 $$MFP = \lambda = \frac{{k_{B} T}}{{p \sqrt 2 \pi d_{g}^{2} }}$$
  where $k_{B}$ is the Boltzmann constant; $d_{g}$ is the molecular diameter and $p$ is the partial pressure.
2. The Knudsen diffusion dominates when the pore size is relatively small, for example, the MFP is comparable or even smaller than the pore sizes. The water molecules move with the collisions between molecules as well as against the pore walls (solid surface). When the pore size is small, however, the former one is negligible. The Knudsen diffusion is given by^31^:
  6 $$D_{K} = 9700r_{e} \sqrt{\frac{T}{M}}$$
  where $D_{K}$ is the molecular diffusivity in ${\text{cm}}^{2} /{\text{s}}$; $r_{e}$ is the mean pore radius in ${\text{cm}}$.
3. The Surface diffusion occurs when the pore size is smaller than MFP, so the molecules diffuse along the pore surfaces (walls). The movement of molecules along the pore walls is due to the succession of adsorption–desorption mechanisms^36^. Surface diffusion does not play a significant role in larger pores (e.g., concrete); nevertheless, it will be taken into account in this investigation for precision purposes. The surface diffusion dominates as the pore size decreases to a few nanometers^43^. Usually, surface diffusion poses greater resistance to water movement than Knudsen diffusion. Thus, surface diffusion is considered for adsorbed water. Hence, the surface diffusion is significant for very low humidity in concrete^4^. The surface diffusion is derived based on the adsorption theory by the Langmuir isotherm^32,44,45^:
  7 $$q_{a} = \frac{{q_{L} p}}{{p + p_{L} }}$$
  where $q_{a}$ is the standard volume of adsorbed moisture per unit mass; $p$ is the partial pressure and $p_{L}$ is the Langmuir pressure. The Langmuir pressure is extremely dependent on the energy or heat of adsorption (Δ $H$) and temperature, given by the following expression^46^:
  8 $$p_{L} = p_{L0} exp \left[ {\frac{{{\Delta }H}}{R T}} \right]$$
  where $p_{L0}$ is a constant of the Langmuir pressure (obtained from adsorption isotherm curve). Because Langmuir adsorption is monolayer adsorption, the moisture coverage on nanopore wall at an equilibrium state ($\theta$) can be defined as the ratio of the adsorption volume to the Langmuir volume:
  9 $$\theta = \frac{p}{{p + p_{L} }}$$
  For the surface diffusivity in nano-pore, a quantitative relationship was derived using the kinetic method by Yang and Chen^47^. The model is considered as the most useful model that was derived theoretically^48^ and is given by:
  10 $$D_{S} = D_{S}^{0} \frac{{\left( {1 - \theta } \right) + \frac{\kappa }{2}\theta \left( {2 - \theta } \right) + \left\{ {H\left( {1 - \kappa } \right)} \right\} \frac{\kappa }{2}\theta^{2} }}{{\left( {1 - \theta + \frac{\kappa }{2}\theta } \right)^{2} }}$$

- $D_{S}^{0}$ is the surface diffusion when the moisture coverage is “0” ${\text{m}}^{2} /{\text{s}}$^38,45^. It depends on the temperature and activation energy which depends on the energy of adsorption^49–52^.
  11 $$D_{S}^{0} = {\Omega }T^{m} \times e^{{\left( { - \frac{E}{R T}} \right)}}$$
  where ${\Omega }$ is a constant that relates the molecular weight in (${\text{cm}}^{2} /{\text{s}} \cdot {\text{K}}^{1/2}$); $m$ is a dimensionless constant; $E$ is the activation energy and given in terms of the adsorption energy as $E = {\Delta }H^{0.8}$^45^; $R$ is the gas constant. The range of $D_{S}^{0}$ varies between $10^{ - 6}$ and $10^{ - 4}$ ${\text{cm}}^{2} /{\text{s}}$ for temperatures ranging from 273 to 303 K^52^. Experimentally, the value of $D_{S}^{0}$ is highly dependent on the activation energy and an error of 5% in the activation energy corresponds to a 50% difference in $D_{S}^{0}$, hence it is extremely difficult to find $D_{S}^{0}$ accurately^40^. Therefore, an average value of the range was assumed in this study, $D_{S}^{0} = 1 \times 10^{ - 5} {\text{cm}}^{2} /{\text{s}}$.
- $\kappa$ is dependent on the ratio of the blocking velocity coefficient of surface molecule ($\kappa_{b}$) to the forward velocity coefficient of surface molecule ($\kappa_{m}$).
  12 $$\kappa = \frac{{ \kappa_{b} }}{{\kappa_{m} }}$$
- $H\left( {1 - \kappa } \right)$ is the Heaviside function, expressed as:
  13 $$H\left( {1 - \kappa } \right) = \left\{ {\begin{array}{*{20}l} {0, \kappa \ge 1} \\ {1, 0 \le \kappa \le 1} \\ \end{array} } \right.$$

As can be clearly noticed from Eq. (13) when surface diffusion occurs, $\kappa$ ranges from $0$ to $1$. That means the forward velocity coefficient $\kappa_{m}$ is larger than the blocking velocity coefficient. If $\kappa_{b}$ is greater than $\kappa_{m}$, surface diffusion will stop. Based on the literature review, here are some selected values for some of the parameters^38,53^: $\kappa = 0.5$; $p_{L0} = 8.45 \times 10^{7} {\text{Pa}}$; ${\Delta }H = 10 {\text{kJ}}/{\text{mol}}$.

## Effective diffusivity model

Considering all three diffusion mechanisms, the effective diffusivity model of the bulk porous material can be given by the volumetric average of the three mechanisms and the weighted average parameters are the pore volume fractions of the three mechanisms:

14 $$D_{e} = \varepsilon_{1} { }D_{M} + \varepsilon_{2} { }D_{K} + \varepsilon_{3} { }D_{S}$$

where $\varepsilon_{i}$ are the connected-pore volume fractions of the three domains of molecular ($D_{M}$), Knudsen ($D_{K}$) and surface ($D_{S}$) diffusion, which can be obtained by using a model for the pore size distribution. Houst and Wittmann^36^ developed Eq. (14), in which, the connected-pore volume fractions were obtained experimentally through MIP test. However, in the current study, a theoretical model for the moisture effective diffusivity will be developed, based on the adsorption isotherms, to obtain the volume fractions of the connected-pore system. As mentioned before, the microstructure of cement paste depends on many factors, so the model should consider important factors such as water-to-cement ratio ($w/c$), age of concrete ($t$), curing temperature ($T$) and pore size distribution. The pore size distribution model will be described in the next section. In addition to the four factors, there are other parameters that are essential to be considered e.g., moisture content inside pores (in terms of pore relative humidity). Hence, an impact function, $F\left( H \right)$, that reflects the influence of moisture content inside pores is included in the proposed model as follows:

15 $$D_{e} = \left( {\varepsilon_{1} D_{M} + \varepsilon_{2} D_{K} + \varepsilon_{3} D_{S} } \right) F\left( H \right)$$

Herein, the contribution of each dominant diffusion mechanism was considered. The ranges of diffusivities are dependent substantially on the size of the pores and the mean free path of water. The values of diffusivities were obtained based on literature review^4,36^, and they can be found along with the corresponding equations in Table 1. It is noteworthy that the effective diffusivity is the humidity diffusivity coefficient $D_{h}$, where the final form of the humidity diffusivity model is given by the composite sphere model because concrete is a composite material therefore, the composite effect considered^54^:

16 $$D_{h} = D_{e} \left[ {1 + \frac{{D_{i} }}{{\frac{{1 - g_{i} }}{3} + \frac{{D_{e} }}{{D_{i} - D_{e} }}}}} \right]$$

where $D_{i}$ is the diffusivity of inclusions, i.e., aggregates and $g_{i}$ is the volume fraction of aggregates^55^.

**Table 1:** Range of pore-sizes, transport mechanism and corresponding diffusion equation.

Range of pores of diameter $d$ ($\lambda$ = MFP; T = 296 K; P = 1 atm)	Transport mechanism	Diffusion equation
$d \ge \lambda$ (H_2_O: $d \ge 86 {\text{nm}}$)	Molecular diffusion	Equation (4)
$0.1\lambda < d < \lambda$ (H_2_O: $8.6 {\text{nm}} < d < 86 {\text{nm}}$)	Knudsen diffusion	Equation (6)
$d \le 0.1\lambda$ (H_2_O: $d \le 8.6 {\text{nm}}$)	Surface diffusion	Equation (10)

## Pore-size distributions

### Adsorption isotherm model

The pore-size distribution can be obtained using the adsorption isotherm of concrete. The Brunauer–Emmett–Teller (BET) model is considered as one of the best isotherm models^56^. This model has been modified by researchers such as the BDDT model^57^, FHH model^58^ and the BSB model^59^. The Brunauer-Skalny-Bodor (BSB) model, the so-called three-parameter BET model, was used to describe the adsorption isotherm of concrete. It is applicable for the humidity (relative pressure) ranges from 0.05 to 1.0. Later on, Xi et al.^34^ established prediction models for the three parameters of the adsorption isotherm, which is based on the BSB model:

17 $$W\left( H \right) = \frac{{ V_{m} C k H}}{{\left( {1 - kH} \right)\left[ {1 + \left( {C - 1} \right)kH} \right]}}$$

where $W\left( H \right)$ is the quantity of vapor adsorbed at equilibrium pressure $p$ in grams of water per gram of cement paste; $H = p/p_{s}$ (which is the pore relative humidity, as defined before), $p_{s}$ is the pressure at saturation; $V_{m}$ is the monolayer capacity; $C$ and $k$ are the other two parameters used in BSB model. $V_{m}$, $C$ and $k$ are the three parameters that can be found using the adsorption isotherm model^34^ :

1. The monolayer capacity, parameter $V_{m}$:
  18 $$V_{m} = V_{ct} \left( {C_{t} } \right) V_{wc} \left( {w/c} \right) V_{t} \left( t \right) V_{T} \left( T \right)$$
  $V_{ct} \left( {C_{t} } \right)$: considers the effect of cement type:
    19 $$V_{ct} = \left\{ {\begin{array}{*{20}l} {0.9, } \hfill & {Type I} \hfill \\ {1.0,} \hfill & {Type II} \hfill \\ {0.85,} \hfill & {Type III} \hfill \\ {0.6,} \hfill & {Type IV} \hfill \\ \end{array} } \right.$$
  $V_{wc} \left( {w/c} \right)$: considers the effect of water-to-cement ratio. For $0.3 \le w/c \le 0.7$:
    20 $$V_{wc} \left( {w/c} \right) = 0.85 + 0.45 \left( {w/c} \right)$$
    When $w/c < 0.3$,$w/c = 0.3$; Similarly, when $w/c > 0.7, w/c = 0.7$.
  $V_{t} \left( t \right)$: considers the effect of curing time, where $t$ is the age of concrete in days. For $t \le 5$, use $t = 5$ days. Otherwise:
    21 $$V_{t} \left( t \right) = 0.068 - \frac{0.22}{t}$$
  $V_{T} \left( T \right)$: considers the effect of temperature ($T$). $V_{m}$ is influenced by thermal contraction and/or expansion. Since the volumetric changes of the water vapor are very small at room temperature then $V_{T} \left( T \right)$ can be taken to be 1.0^34^.
2. Parameter $$C$$:
  22 $$C = exp\left( {\frac{{C_{0} }}{T}} \right){ }$$
  where $C_{0}$ is obtained from test data and was found to be $855$^34^.
3. Parameter $k$ is a constant such that $0 < k < 1$; it can be calculated using the following formula:
  23 $$k = \frac{{\left( {1 - \frac{1}{n}} \right)C - 1}}{{\left( {C - 1} \right)}}$$
  where $n$ is the number of adsorbed layers at the saturation state. Since $0 < k < 1$, then $n > 1$^34^. The parameter $n$ can be obtained similarly to parameter $V_{m}$:
  24 $$n = N_{ct} \left( {C_{t} } \right) N_{wc} \left( {w/c} \right) N_{t} \left( t \right) N_{T} \left( T \right)$$

- $N_{ct} \left( {C_{t} } \right)$: considers the effect of cement type:
  25 $$N_{ct} = \left\{ {\begin{array}{*{20}l} {1.1,} \hfill & {Type I} \hfill \\ {1.0,} \hfill & {Type II} \hfill \\ {1.15,} \hfill & {Type III} \hfill \\ {1.5, } \hfill & {Type IV} \hfill \\ \end{array} } \right.$$
- $N_{wc} \left( {w/c} \right)$: considers the effect of water-to-cement ratio. For $0.3 \le w/c \le 0.7$:
  26 $$N_{wc} \left( {w/c} \right) = 0.33 + 2.2 \left( {w/c} \right)$$
  When $w/c < 0.3$,$w/c = 0.3$; Similarly, when $w/c > 0.7, w/c = 0.7$.
- $N_{t} \left( t \right)$: considers the effect of curing time, where $t$ is the age of concrete in days. For $t \le 5$, use $t = 5$ days; otherwise:
  27 $$N_{t} \left( t \right) = 2.5 + \frac{15}{t}$$
- $N_{T} \left( T \right)$: considers the effect of temperature ($T$). $N_{m}$ is influenced by thermal contraction and/or expansion. Since the volumetric changes of the water vapor are very small at room temperature then $N_{T} \left( T \right)$ can be taken to be 1.0^34^.

This model encompasses the typical range of concrete design parameters, including a water-to-cement (w/c) ratio from 0.3 to 0.7, ASTM C150 Types I, II, III, and IV cements, and an aggregate volume fraction from 0.5 to 0.8. Moreover, the model is applicable to ambient temperature conditions.

### Development of pore size distribution function ${\varvec{G}}\left( {\varvec{x}} \right)$

In this section, the distribution function for pores inside concrete is explained. Jaroniec and Choma model^60^ was used to find the pore-size distribution function for concrete with the help of the adsorption isotherm model Eq. (17). Jaroniec and Choma’s approximation for $W\left( H \right)$ is used to find the distribution function. The pore-size distribution function $G\left( x \right)$ is given by Eq. (28):

28 $$G\left( x \right) = \frac{{(q/c)^{m + 1} }}{{\Gamma \left( {m + 1} \right)}}x^{2m + 1} {\text{exp}}\left( { - qx^{2} /c} \right)$$

where $x$ is the micropore dimension (pore size); $\Gamma$ is the Gamma function; c is an adjustable parameter to make sure of the unity of the area under the pore-size distribution function. $\Delta$ is the integration region which is between $\left( {0, \infty } \right)$. $G\left( x \right)$ is obtained by solving the integration of Eq. (29):

29 $$W\left( H \right) = \mathop \smallint \limits_{\Delta }^{ } exp [ - \left( {x/c)^{2} y} \right] G\left( x \right) dx$$

According to Jaroniec and Choma’s approximation, the best curve fit model that provides an excellent representation of molecular adsorption data on microporous solids is found in Eq. (30):

30 $$W\left( H \right) = W_{0} \left( {\frac{q}{q + y} } \right)^{m + 1}$$

where $q,m$ and $W_{0}$ are parameters that can be found by curve fitting, keeping in mind that $W_{0} , q > 0,$ and $m > - 1$^61,62^. Regarding the constant c, it changes based on the given data to satisfy $\mathop \smallint \limits_{z}^{\infty } G\left( x \right) dx = 1$, different values of z would result in different values of c. A value of 211,300 is selected, which represents the c-value when $w/c$ ratio is $0.5$, $t$ is $56$ days and z starts at approximately 0.

Parameter $y$ is given by the following formula:

31 $$y = \left( {R T\frac{{{\text{ln}}\left( {p/ps} \right) }}{\beta }} \right)^{2}$$

where $\beta$ is the affinity coefficient that depends on the adsorbate. The ranges for affinity coefficient of water to benzene ($\beta_{w/benzene}$) is 0.07 to 0.26^63–65^. Based on previous literature, $\beta_{benzene}$ was found to be equal to unity^60–62^. Therefore, $\beta$ for vapor water is taken to be 0.1 in this study which also lies within the given range (0.07–0.26).

**Figure 2:**
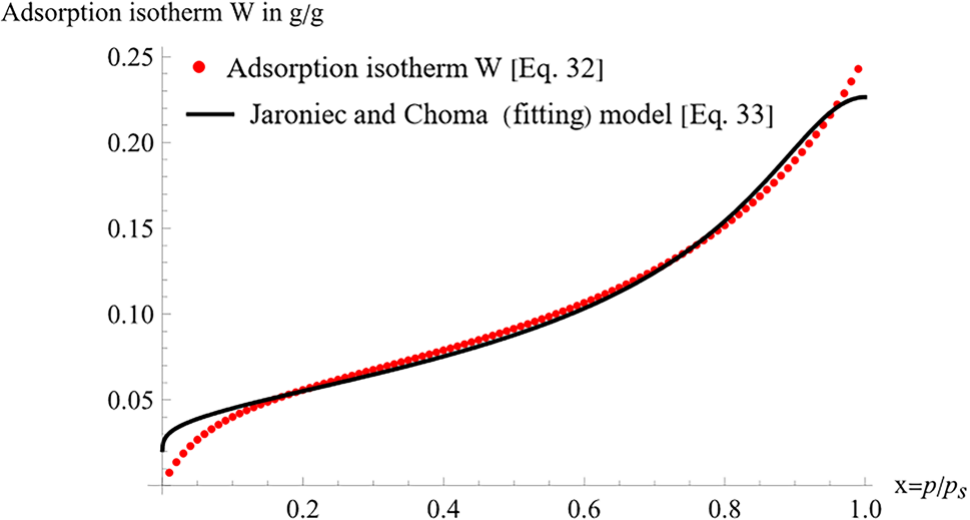
Adsorption isotherm for concrete.

**Figure 3:**
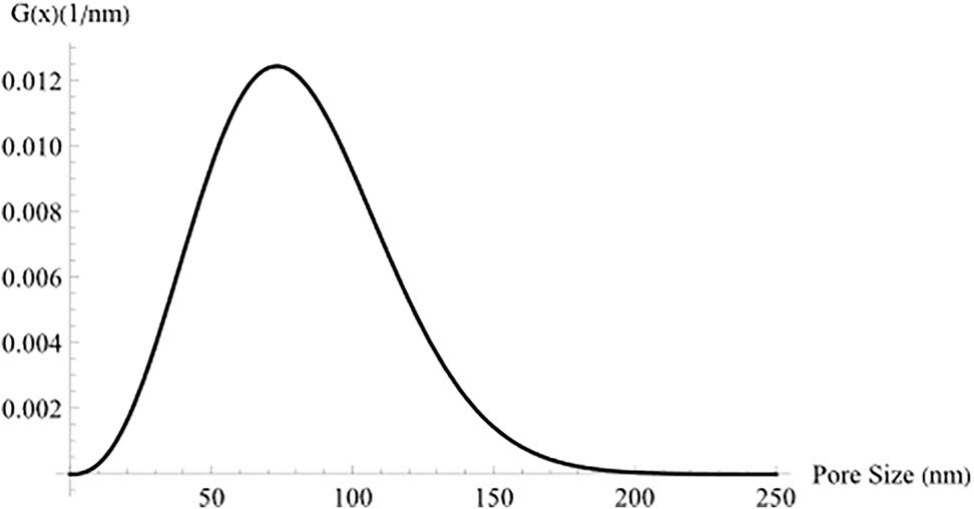
The pore-size distribution, based on the adsorption isotherm.

A brief numerical example is provided here to show how to find the pore size distribution function $G\left( x \right)$ by using the adsorption isotherm model^34^ and the approximation model^60^. Considering a concrete of cement type I, water-to-cement ratio of 50% and curing time of 56 days. the moisture content functions $W\left( H \right)$ obtained from Eq. (32) and the fitted model of Eq. (33) are plotted in Fig. 2. The adsorption isotherm of Eq. (17) and the fitting model of Eq. (30) were solved using Mathematica^66^, and the fitting model was obtained by means of the built-in function “Nonlinearfit” taking into consideration all constraints, as explained in^67^. As can be clearly seen from Fig. 2, the fitting model accurately matches the Jaroniec and Choma’s moisture content model. Moreover, the c value was calculated and found to be approximately 211,300. The distribution function $G\left( x \right)$ was obtained using Eq. (28) and plotted in Fig. 3. As can be noticed, at saturation, the water content is 0.2264. Furthermore, the fitting model was checked with another study conducted by Sicat and Ueda^68^, in which the maximum water content was found to be 0.228. With an error of 0.7%, it can be concluded that the fitting model is extremely precise.

32 $$W\left( {p/p_{s} } \right) = \frac{0.851 H}{{\left( {1.{0} - 0.757H} \right)\left( {1.0 + 12.976H} \right)}}$$

33 $$W\left( {p/p_{s} } \right) = 0.22644\left( {\frac{{101.809 \times 10^{3} }}{{101.809 \times 10^{3} + y}}} \right)^{{\left( { - 0.71957 + 1} \right)}}$$

From the distribution function $G\left( x \right)$ and the diffusion mechanisms limits (Table 1), one can obtain the pore size volume fractions for molecular, Knudsen and surface diffusion mechanisms.

### Pore volume fractions

**Figure 4:**
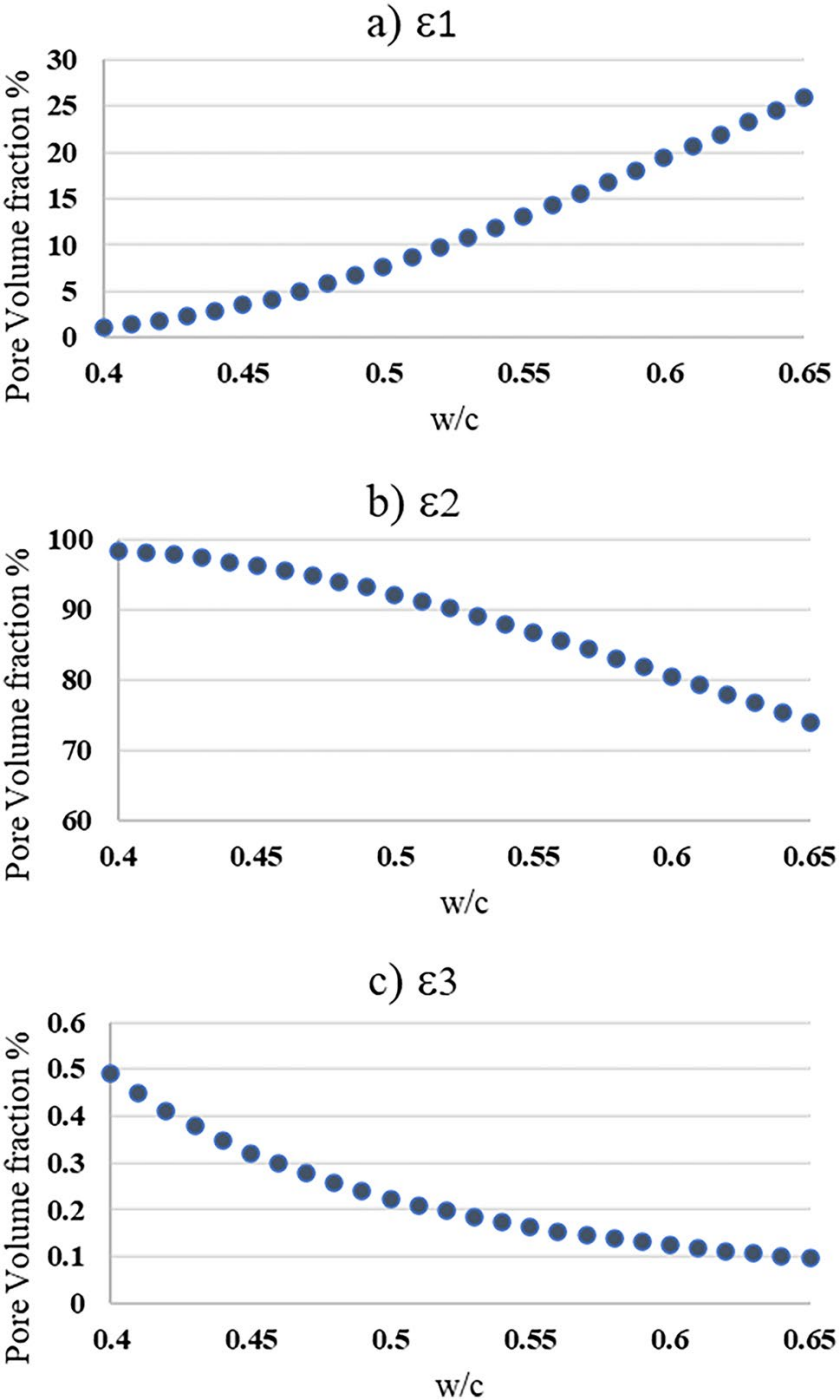
Pore volume fractions at different w/c ratios: (**a**) Molecular diffusion ($\varepsilon_{1}$), (**b**) Knudsen diffusion ($\varepsilon_{2}$) and (**c**) Surface diffusion ($\varepsilon_{3}$).

The pore volume fractions ($\varepsilon_{i}$) for the three domains, i.e., the molecular ($\varepsilon_{1} )$, Knudsen ($\varepsilon_{2} )$ and surface ($\varepsilon_{3}$) diffusions, were obtained by the integration of the pore size distribution function $G\left( x \right)$ over the stated pore size limits in Table 1. The effect of $w/c$ ratio and curing time on pore volume fractions was studied. As can be seen from Fig. 4, the different pore volume fractions were plotted against $w/c$ ratio ranging from 0.4 to 0.65. For the pore volume fraction that is contributed from the molecular diffusion ($\varepsilon_{1}$), as the $w/c$ ratio increases, the larger the molecular pore volume fraction. This is expected because with increasing w/c more large pores exist. Furthermore, from Fig. 4b, most of the pores fall within the Knudsen diffusion range, which means that the Knudsen diffusion mechanism exploits the largest portion of concrete pores. Also, Fig. 4c, shows that the surface diffusion reduces as the water-to-cement ratio increases. This is the expected behavior because as $w/c$ increases, more larger pores will appear in the microstructure.

The formation of pores is significantly influenced by the hydration rate of cementitious materials, which consequently affects the pore volume fractions. The development of pore volume fractions were considered using Xi and Jennings model^69^ with the help of Taylor^70^, see Eq. (34), to obtain the degree of hydration for each reacting compound ($C_{3} S,{ }C_{2} S,{ }C_{3} A,{ }C_{4} AF$).

34 $$\alpha_{i} = 1 - \exp \left[ { - a_{i} \left( {t - b_{i} } \right)^{{c_{i} }} } \right]$$

where $\alpha$ denotes the degree of hydration of each compound in the cement paste, calculated as a weighted average; $t$ denotes the time in days; the coefficients $a_{i}$, $b_{i}$ and $c_{i}$ are specified in Table 2 below.

**Table 2:** Coefficients of the reacting compounds.

Compound	$a_{i}$	$b_{i}$	$c_{i}$
C3S	0.25	0.9	0.7
C2S	0.46	0	0.12
C3A	0.28	0.9	0.77
C4AF	0.26	0.9	0.55

**Figure 5:**
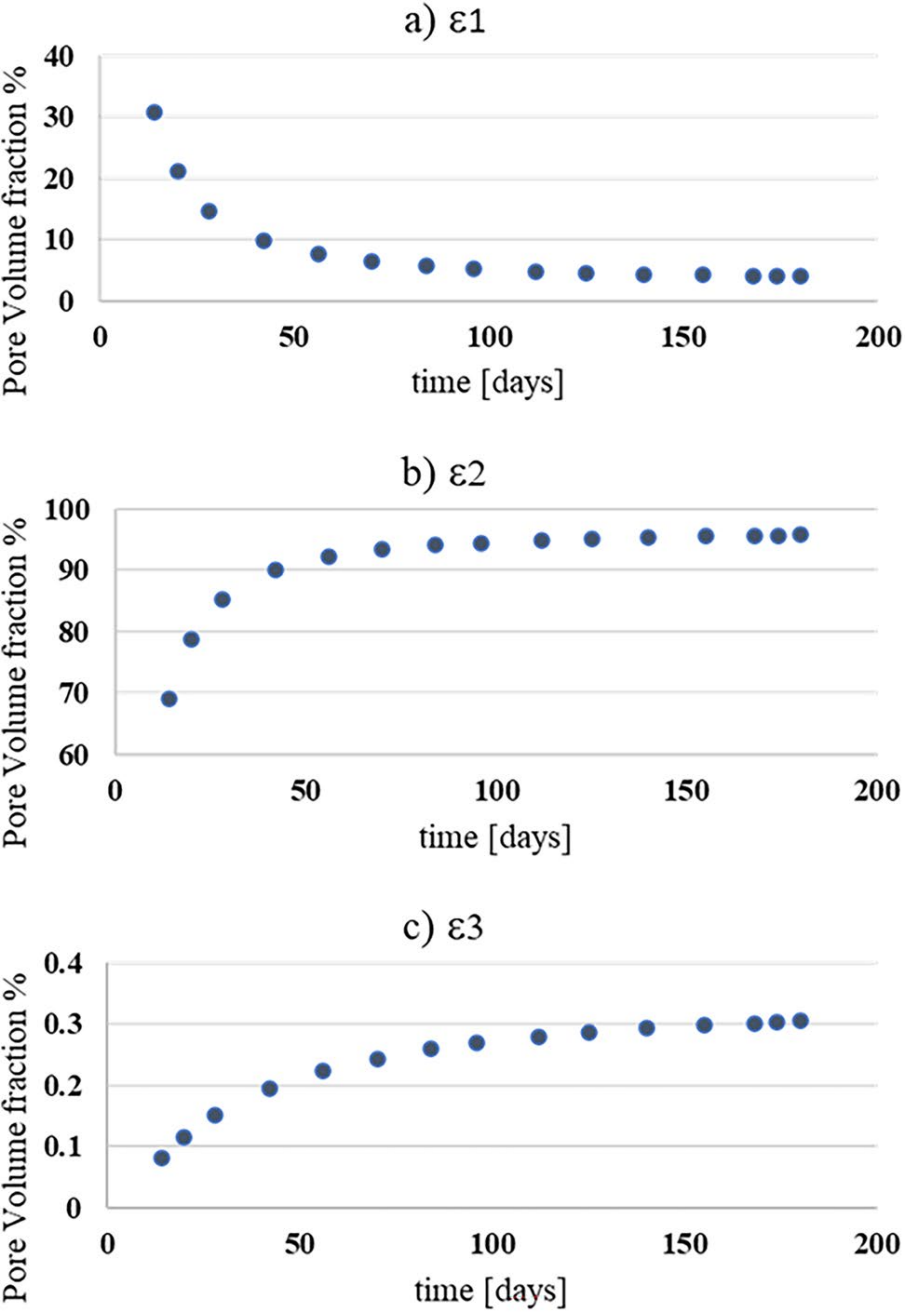
Pore volume fractions variation with time: (**a**) Molecular diffusion ($\varepsilon_{1}$), (**b**) Knudsen diffusion ($\varepsilon_{2}$), (**c**) Surface diffusion ($\varepsilon_{3}$).

The effect of curing time on pore volume fractions was investigated. As can be seen in Fig. 5a, $\varepsilon_{1}$ decreases as the curing time increases. The more the concrete is cured, the less larger pores can be found, hence less molecular pore volume fraction. Conversely, the Knudsen and Surface diffusion pore volumes would increase as can be seen in Fig. 5b,c. These mean that increasing the concrete age, the microstructure develops more and the pore sizes decrease making the Knudsen and surface diffusion as control mechanisms.

### Moisture diffusion analysis

In order to compare the model prediction with available test data, the proposed model for concrete moisture diffusivity was utilized in a finite element analysis of moisture diffusion using MOOSE framework. As explained previously, the adsorption isotherm of concrete will be utilized to obtain the moisture content $W\left( H \right)$ in concrete using the models for the isotherm parameters. Then the pore size distribution function will be determined, and the pore volume fractions will be obtained by integration using Mathematica^66^. The MOOSE framework was utilized to solve the governing equation Eq. (3), in which, the weak form of the governing equation is required^3^ to find the unknowns (relative humidity inside concrete). Preconditioned Jacobian-Free Newton Krylov method (PJFNK) was adopted to solve the nonlinear equations^71,72^. The problem was analyzed at a 2D level using quadrilateral elements and a mesh size of 0.01 cm, a similar model was utilized by Dehwah and Xi^73^.

**Figure 6:**
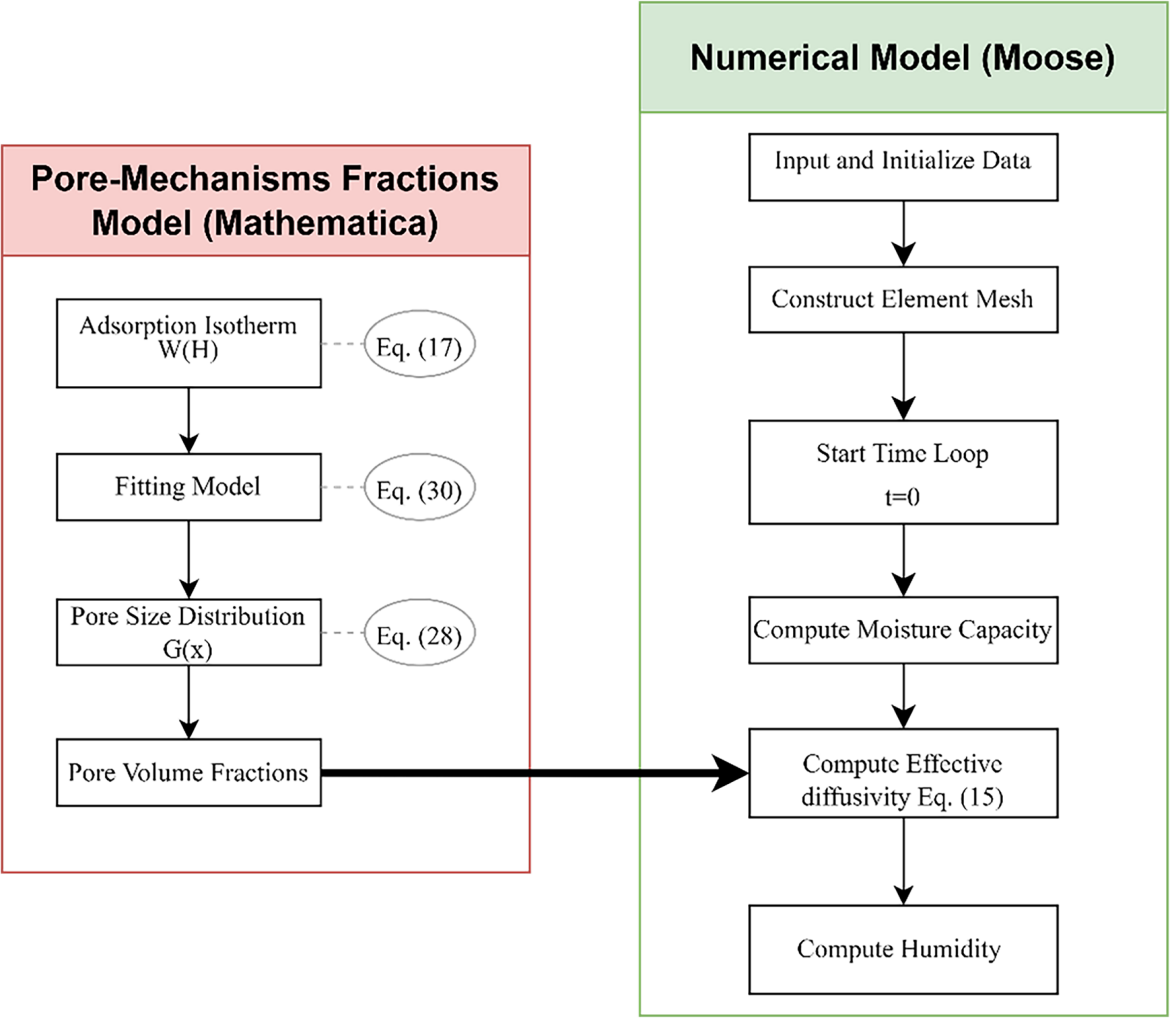
Schematic illustration of the proposed model, (Flow chart summary).

A detailed schematic illustration of the proposed methodology is shown in Fig. 6. The flowchart explains how the proposed model was developed and the associated equations. The results of the model are very accurate for the moisture content inside concrete as explained in section “Development of pore size distribution function”. Also, the relative humidity profiles are considered to be satisfactory as will be illustrated in the next section.

## Effective moisture diffusivity model validation

**Figure 7:**
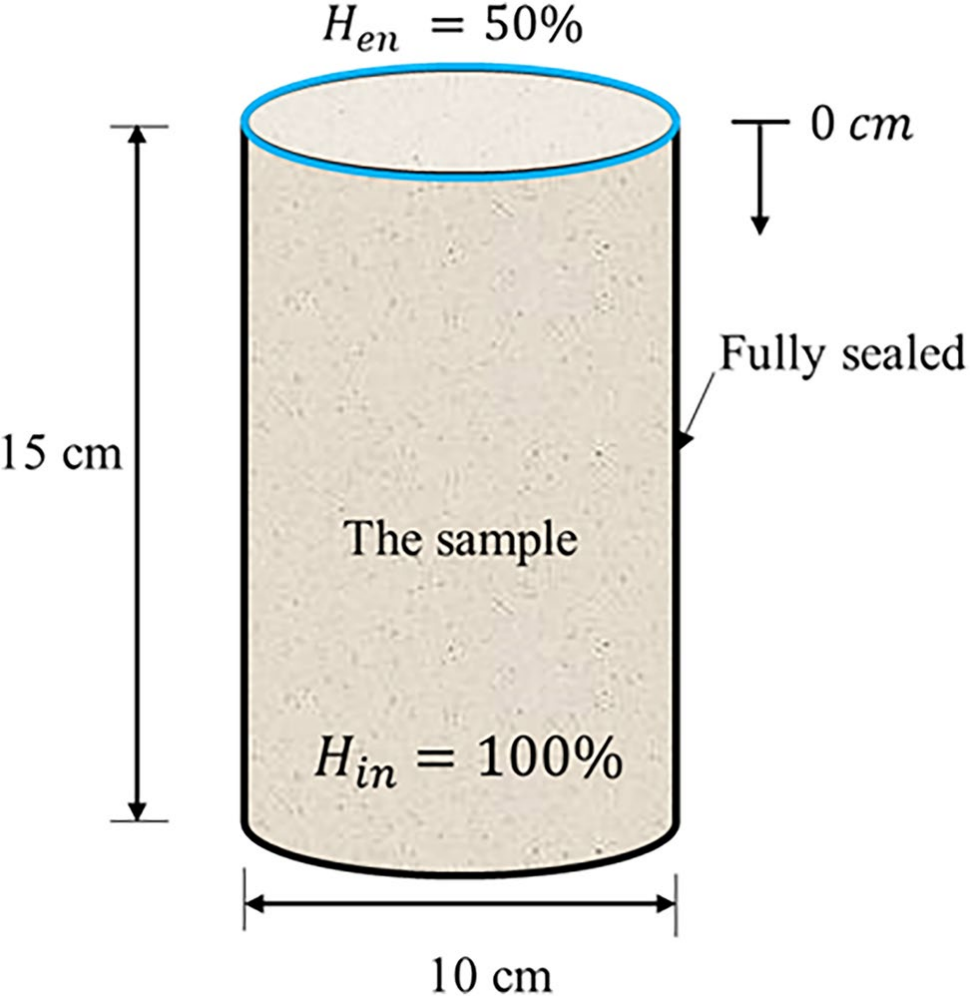
Relative humidity test setup.

The effective moisture diffusivity model was validated with experimental results conducted by Xi et al.^4^ and Kim and Lee^74^. The first experiment^4^ utilized cylindrical concrete samples measuring 15 cm in length, and in an environmental relative humidity ($$H_{en} )$$ of 50%. Samples were cast with two water-to-cement ratios of 0.5 and 0.63, and subsequently cured for three days. Initially, the samples were fully saturated ($$H_{in}$$). Figure 7 shows the sample along with the humidity boundary conditions. Moreover, the second experiment^74^ was conducted on a cubic sample of the following dimensions $$10 {\text{cm}} \times 10 {\text{cm}} \times 20 {\text{cm}}$$ under similar condition, however, the water-to-cement ratio was 0.68. Table 3 provides a summary of the three samples that were tested experimentally. The samples were fully sealed from all sides except the top side; hence no humidity dissipation was allowed except from the top surface.

**Table 3:** Summary of experimental samples properties.

Sample #	w/c	Curing time (days)	$H_{en}$(%)	$H_{in}$(%)	Length (cm)
1^4^	0.5	3	50	100	15
2^4^	0.63	3	50	100	15
3^74^	0.68	3	50	100	20

**Figure 8:**
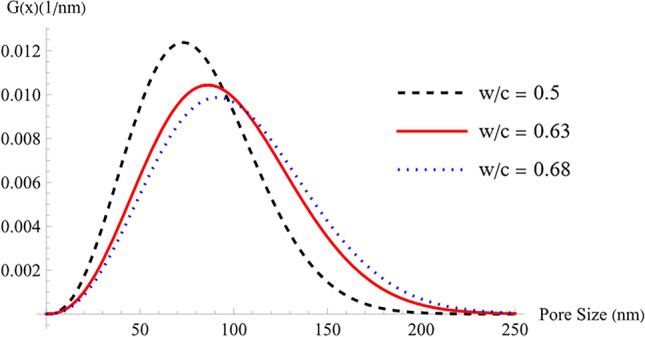
Pore size distribution.

The pore size distributions of the three samples are illustrated in Fig. 8, using the developed adsorption isotherm model. Observations reveal that the sample with a higher water-to-cement (w/c) ratio exhibits a broader size distribution, suggesting the existence of larger voids facilitating increased molecular diffusion. Consequently, this sample possesses a greater volume fraction for molecular diffusion.

**Figure 9:**
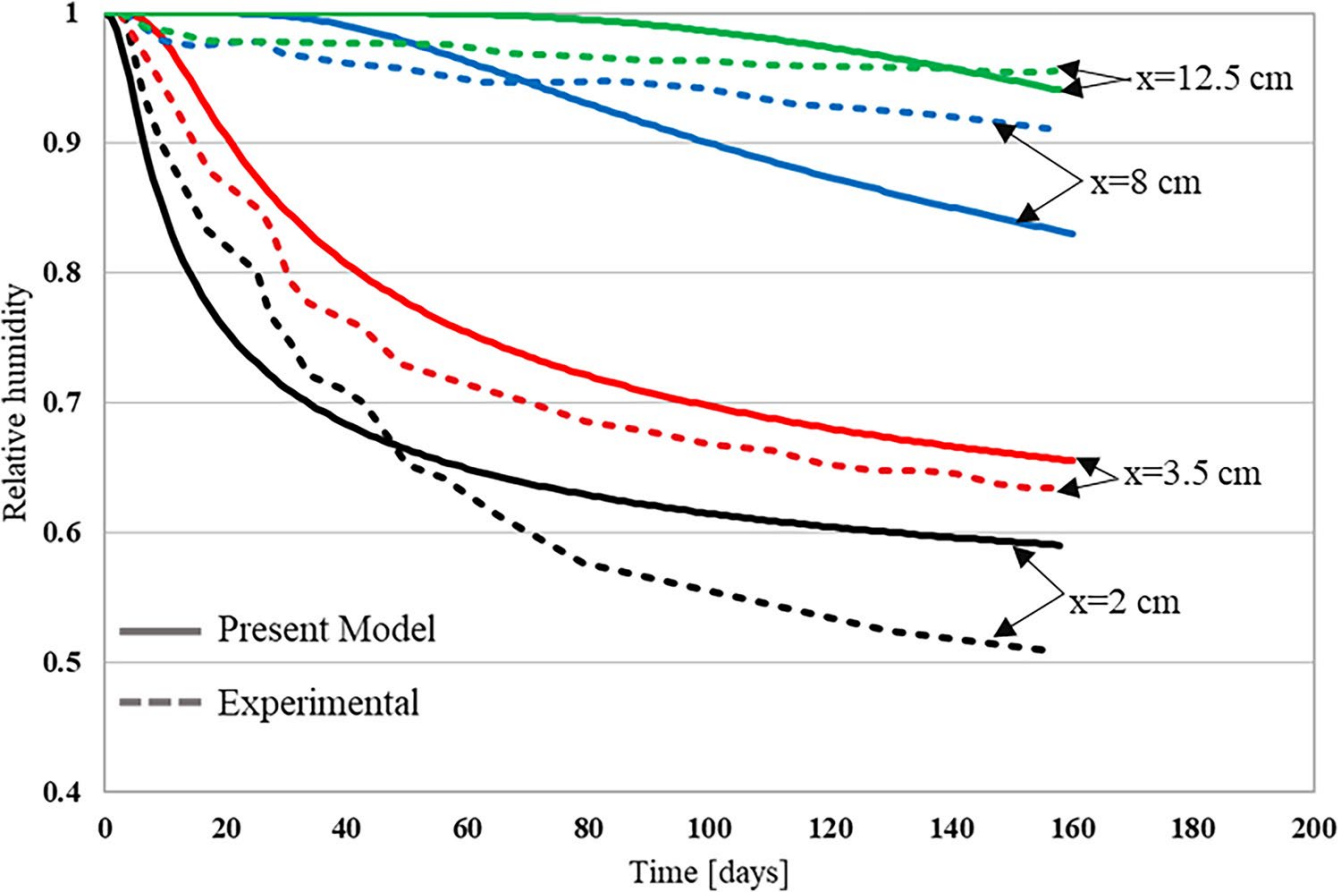
Relative humidity profile for w/c of 0.5^4^.

**Figure 10:**
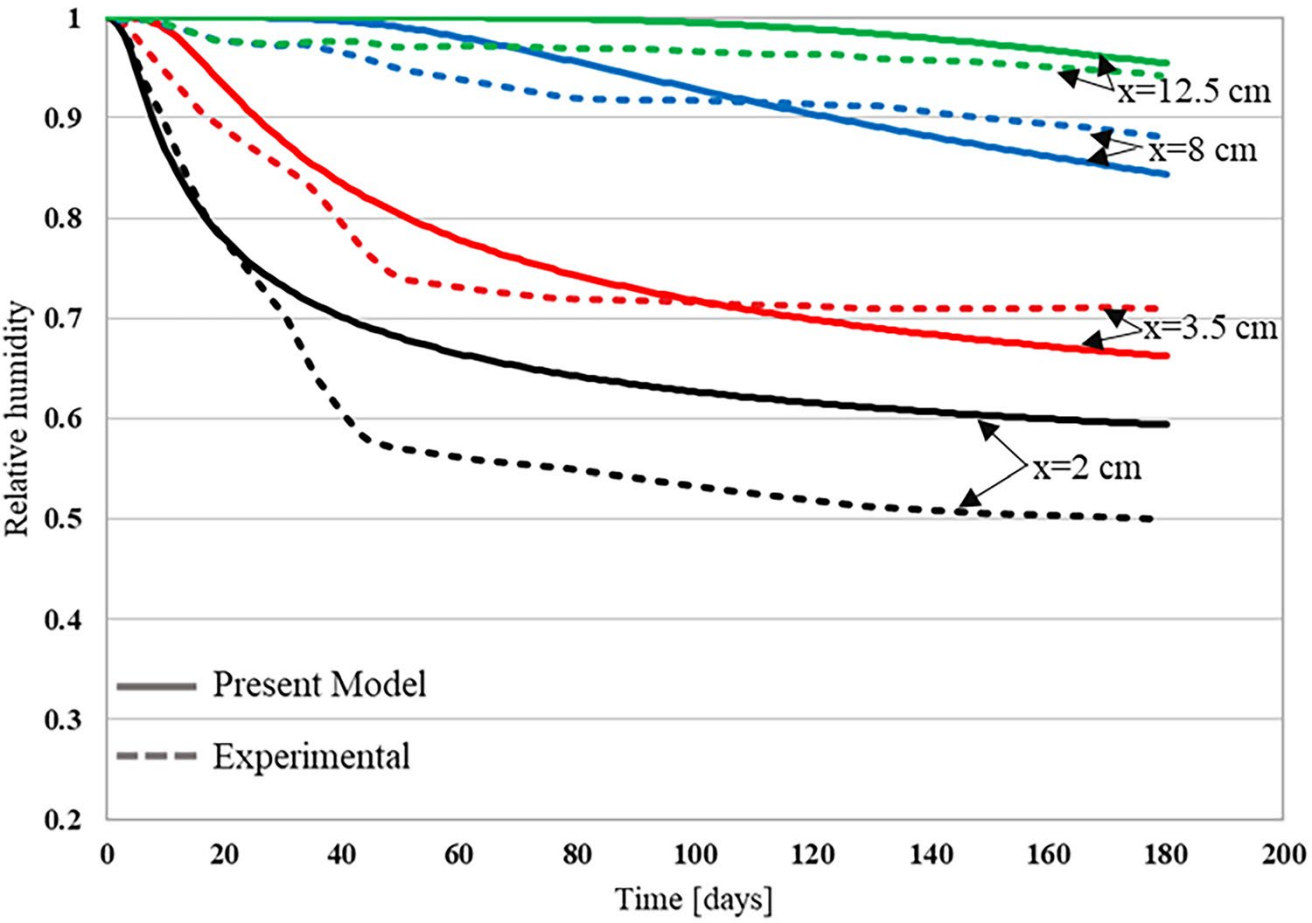
Relative humidity profile for w/c of 0.63^4^.

**Figure 11:**
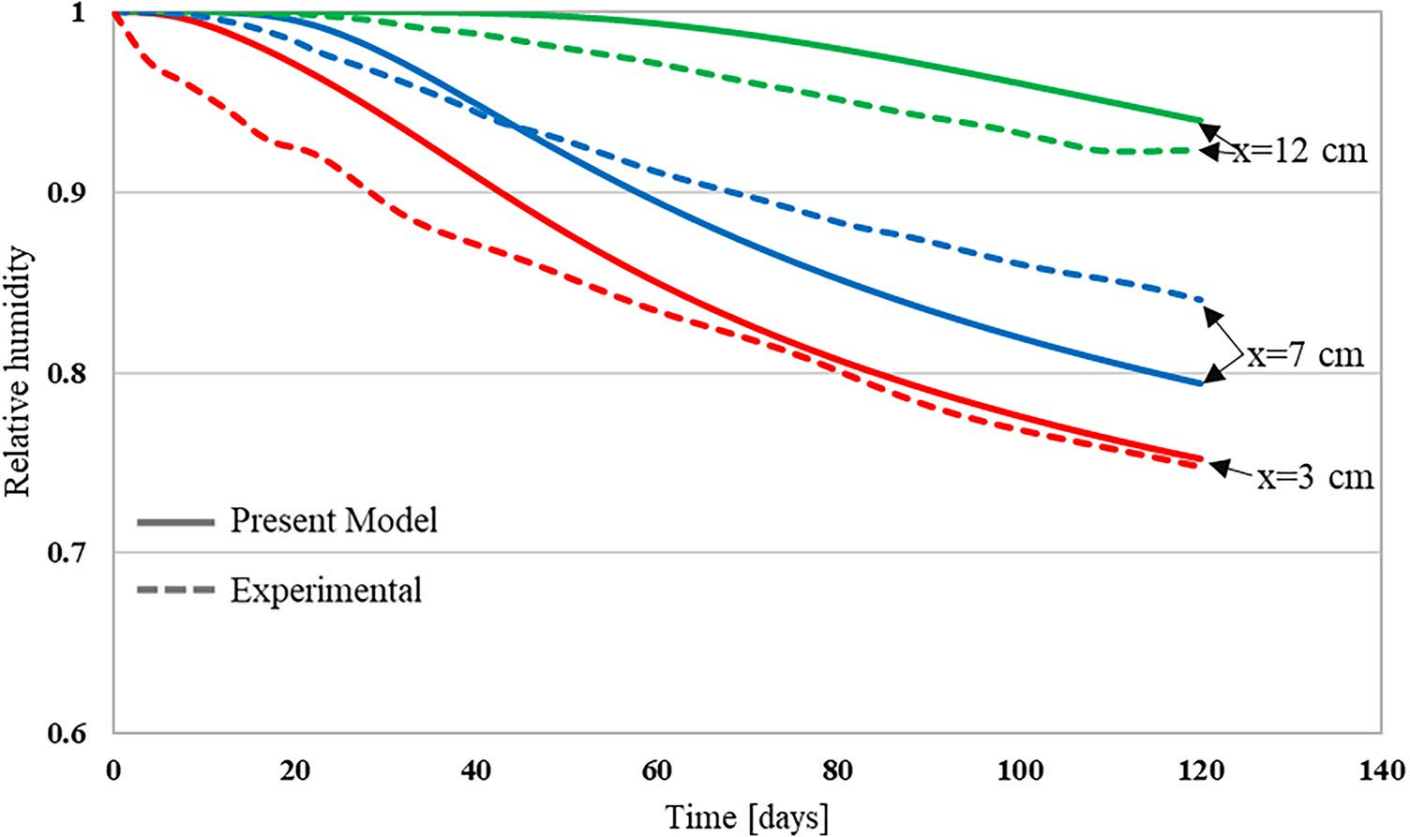
Relative humidity profile for w/c of 0.68^74^.

For Xi et al.^4^ experiment, the time dependent relative humidity profiles were determined at different depths of $2$ ${\text{cm}}$, $3.5$ ${\text{cm}}$, $8$ ${\text{cm}}$ and $12.5$ ${\text{cm}}$. The sample depth is zero at the top and 15 cm at the bottom. Figures 9 and 10 show a comparison between the experimental relative humidity profiles and the numerical ones obtained by the proposed approach that was implemented using the finite element code MOOSE, for the two sample categories. As can be seen from both figures, the relative humidity decreases as the time increases. The initial saturation of samples combined with the low environmental relative humidity induced the drying of concrete. The surface concrete layer exhibits a sharp drop in relative humidity while the deeper layer exhibits a smooth and slow transition from high to low relative humidity. This is due to the fact that the moisture flux is very high near the surface, as large relative humidity gradients exist at the concrete surface. The deeper the moisture moves inside concrete the slower it becomes, as fluxes will be reduced because of the reduced relative humidity gradients. The simulated humidity profiles using the developed moisture diffusivity model successfully matched the trend of the experimental results. Table 4 presents a comparative analysis between the experimental results and the predictions of the developed model, particularly focusing on errors at various depths. The maximum errors were observed at a depth of 2 cm, near the surface, when compared to the deeper concrete regions. This is because the volume fraction of large pores is higher near the surface, which means that the molecular diffusion is the dominating mechanism. As the pore size distribution function gives an average of each pore size volume fraction, it may underestimate the volume fraction of large pores near the surface. Consequently, the contribution of the molecular diffusion to the global amount of transported moisture will be underestimated. Hence, the drying of concrete will be slower at the surface, and the resulting relative humidity profile will be higher than the experimental one. Moreover, for water-to-cement ratios (w/c) of 0.5 and 0.63, the average relative errors were 5.8% and 12.5%, respectively. Meanwhile, R^2^ values of 0.94 and 0.97, obtained using linear regression for each respective ratio, indicated a strong fit of the developed model. Errors at other depths consistently remained below 10%, suggesting a reliable performance of the model for various conditions considered in this study. Similar results were obtained from the experiment conducted by Kim and Lee^74^, where relative humidity was measured at three different depths: 3 cm, 7 cm, and 12 cm, as shown in Fig. 11. Among these depths, the maximum error was found to be approximately 5% at a depth of 3 cm and at 20 days. These findings imply that the developed model can be considered realistic and practical within the tested range.

**Table 4:** Comparison of developed model results with experimental.

Depth (cm)	w/c 0.5		w/c 0.63	
	Maximum relative error (%)	Average relative error (%)	Maximum relative error (%)	Average relative error (%)
2	16	5.8	19.6	12.5
3.5	8.0	4.3	9.1	4.9
8	7.5	3.5	7.3	3.4
12.5	2.9	1.8	3.6	2.5

To capture the effect of moisture level on the moisture diffusivity, an empirical formula was proposed for the impact function $F\left( H \right)$ (Eq. (15)). It was found to be simple and linear as follows:

35 $$F\left( H \right) = 11.53 H + 80.7$$

This function accounts for the influence of the relative humidity and was calibrated based on experimental data. With increasing moisture level, the function $F\left( H \right)$ increases, meaning the resistance to moisture transfer will be lower. The other factors that affect the concrete microstructure such as w/c ratio, curing time, type of cement and temperature, were already taken into account in the model parameters for the adsorption isotherms.

## Conclusions

The transport rate of aggressive materials into concrete has a strong influence on the durability of concrete. Moisture diffusivity plays an important role in determining the rate and amount of transported species in concrete. The moisture diffusivity is affected by the concrete microstructure, namely the pore size distribution, which varies from the nanometer scale to the millimeter scale. The concrete microstructure is influenced by many fundamental parameters such as the water-to-cement ratio, the curing time, the cement type and the temperature. In this paper, a theoretical model for estimating the moisture diffusivity in concrete was developed. The proposed approach was based on a combination of the diffusion mechanisms, namely the molecular, Knudsen and surface diffusions. The contribution of each diffusion mechanism was assessed and accounted for by means of a weighting parameter that is directly dependent on the corresponding pore size volume fraction. The effects of the water-to-cement ratio, type of cement, curing time, and temperature were also considered. The developed model was then used in a finite element code to assess the moisture transport in concrete. The obtained relative humidity profiles were validated against available experimental data. The outcomes of the present study are:

- The developed theoretical model was able to reflect the influence of the water-to-cement ratio and the age of concrete on the rate of moisture transport. The moisture content obtained was precise with a relative error of 0.7%.
- An empirical impact function that reflects the influence of the relative humidity on the moisture diffusivity was proposed. This function has a linear form with two constants determined based on the experimental data.
- The relative humidity profiles simulated using the proposed model were satisfactorily able to match the existing experimental data. The relative errors, maximum and average, were found to be less than 10% for depths deeper than the surface ($2 {\text{cm}}$), nevertheless the overall $R^{2}$ was found to be greater than or equal to 0.94.

## Data Availability

The datasets generated during and/or analysed during the current study are available from the corresponding author on reasonable request.
